# With a Flick of the Lid: A Novel Trapping Mechanism in *Nepenthes gracilis* Pitcher Plants

**DOI:** 10.1371/journal.pone.0038951

**Published:** 2012-06-13

**Authors:** Ulrike Bauer, Bruno Di Giusto, Jeremy Skepper, T. Ulmar Grafe, Walter Federle

**Affiliations:** 1 Department of Plant Sciences, University of Cambridge, Cambridge, United Kingdom; 2 Department of Biology, University Brunei Darussalam, Gadong, Brunei Darussalam; 3 English Language Center, Ming Chuan University, Taipei, Taiwan; 4 Department of Physiology, Development and Neuroscience, University of Cambridge, Cambridge, United Kingdom; 5 Department of Zoology, University of Cambridge, Cambridge, United Kingdom; University of Northampton, United Kingdom

## Abstract

Carnivorous pitcher plants capture prey with modified leaves (pitchers), using diverse mechanisms such as ‘insect aquaplaning’ on the wet pitcher rim, slippery wax crystals on the inner pitcher wall, and viscoelastic retentive fluids. Here we describe a new trapping mechanism for *Nepenthes gracilis* which has evolved a unique, semi-slippery wax crystal surface on the underside of the pitcher lid and utilises the impact of rain drops to ‘flick’ insects into the trap. Depending on the experimental conditions (simulated ‘rain’, wet after ‘rain’, or dry), insects were captured mainly by the lid, the peristome, or the inner pitcher wall, respectively. The application of an anti-slip coating to the lower lid surface reduced prey capture in the field. Compared to sympatric *N. rafflesiana*, *N. gracilis* pitchers secreted more nectar under the lid and less on the peristome, thereby directing prey mainly towards the lid. The direct contribution to prey capture represents a novel function of the pitcher lid.

## Introduction

Carnivorous pitcher plants have recently emerged as a model system for studying the evolution of functional traits in plant morphology in an ecological context [1]–[7]. Members of the paleotropical genus *Nepenthes* capture prey with specialised, highly modified leaves (pitchers) acting as passive pitfall traps [8]. Most species produce two morphologically distinct pitcher types: ‘lower’ pitchers that usually rest on the ground and develop on immature rosette plants, and hanging ‘upper’ pitchers on climbing vines. Each pitcher consists of the main pitcher body, partly filled with digestive fluid, a collar-like upper rim (peristome), and the pitcher lid which in most species forms a ‘roof’ above the pitcher opening, protecting the pitcher from being flooded by rain.

Pitchers of all *Nepenthes* species secrete nectar to attract insect prey [8]. Extrafloral nectaries are scattered across the outside of the pitcher and both the upper and lower lid surface, and are densely packed around the inner margin of the peristome. The quantity of nectar secreted on different parts of the pitcher (and other parts of the plant) varies with pitcher development, and between species [9], [10]. In fully developed, open pitchers (i.e. functional traps) the largest quantities of nectar are secreted on the peristome and under the pitcher lid [9].

A number of distinct trapping mechanisms have been described, such as specialised slippery surfaces on the peristome [11] and the inner pitcher wall [12], [13], as well as viscoelastic pitcher fluids [14]. The peristome is highly wettable and under humid conditions, thin stable water films form on the surface, rendering it extremely slippery [11]. Due to its wetness-dependence, the peristome only activates the trap intermittently, and visiting insects can safely harvest nectar during inactive (i.e. dry) times [15]. By this means, the plant may promote the survival of ‘scout’ ants that ultimately recruit larger numbers of worker ants to the trap.

**Figure 1:**
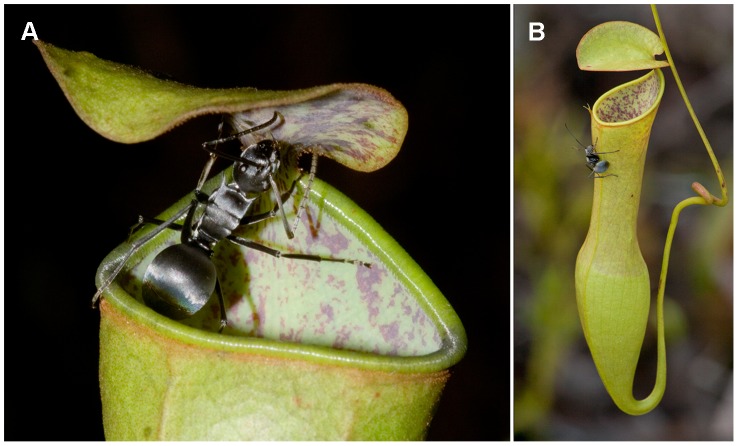
Morphology of *N. gracilis* pitchers. (A) *N. gracilis* pitcher with visiting *Polyrhachis pruinosa* ant, showing the epicuticular wax crystal surfaces on the inner pitcher wall and on the underside of the pitcher lid. (B) The horizontal orientation directly above the pitcher opening puts the lower lid surface in an ideal position for prey capture.

The slipperiness of the inner wall is based on a dense layer of platelet-shaped wax crystals that are orientated perpendicularly to the surface. These crystals drastically reduce the available contact area for insect adhesive pads [16]. In addition, the platelets have been reported to break off easily and contaminate the insects' adhesive pads [13], [17]. The wax crystal layer is a common feature of many *Nepenthes* although there are several species in which it is reduced or absent [5], [6]. *N. gracilis* is unusual in that it has wax crystals not only on the inner pitcher wall but also on the underside of the pitcher lid (Fig. 1A). This characteristic prompted us to investigate whether the lid is involved in prey capture in this species.

We observed that ants harvesting nectar from the lower lid surface of *N. gracilis* in the field (in Brunei, Northern Borneo) were able to walk upside down on the wax crystal surface without difficulty, while the same ants would slip and fall from the waxy inner pitcher wall. Nevertheless, the presence of such a unique structure strongly suggested a trapping function. A casual observation of a Coccinellid beetle being flicked into a *N. gracilis* pitcher by a raindrop after seeking shelter under the pitcher lid prompted us to hypothesise that the wax crystal layer, while providing a secure foothold under normal conditions, causes insects to detach more easily under sudden impacts. The horizontal orientation of the lid directly above the pitcher opening (Fig. 1B) and its comparatively high stiffness could further aid this trapping function. Field observations of increased prey numbers in *N. gracilis* pitchers after rainy days (C. Clarke, personal communication) support this idea; however, they might as well be due to the increased trapping efficiency of the peristome under wet conditions. We therefore investigated the role of the *N. gracilis* lid for prey capture in the laboratory and in the field, and characterised the detailed structure of the wax crystal surface using scanning electron microscopy (SEM).

## Results

### The impact of heavy ‘rain’ drops causes ants to fall from the lower lid surface of *N. gracilis*

**Figure 2:**
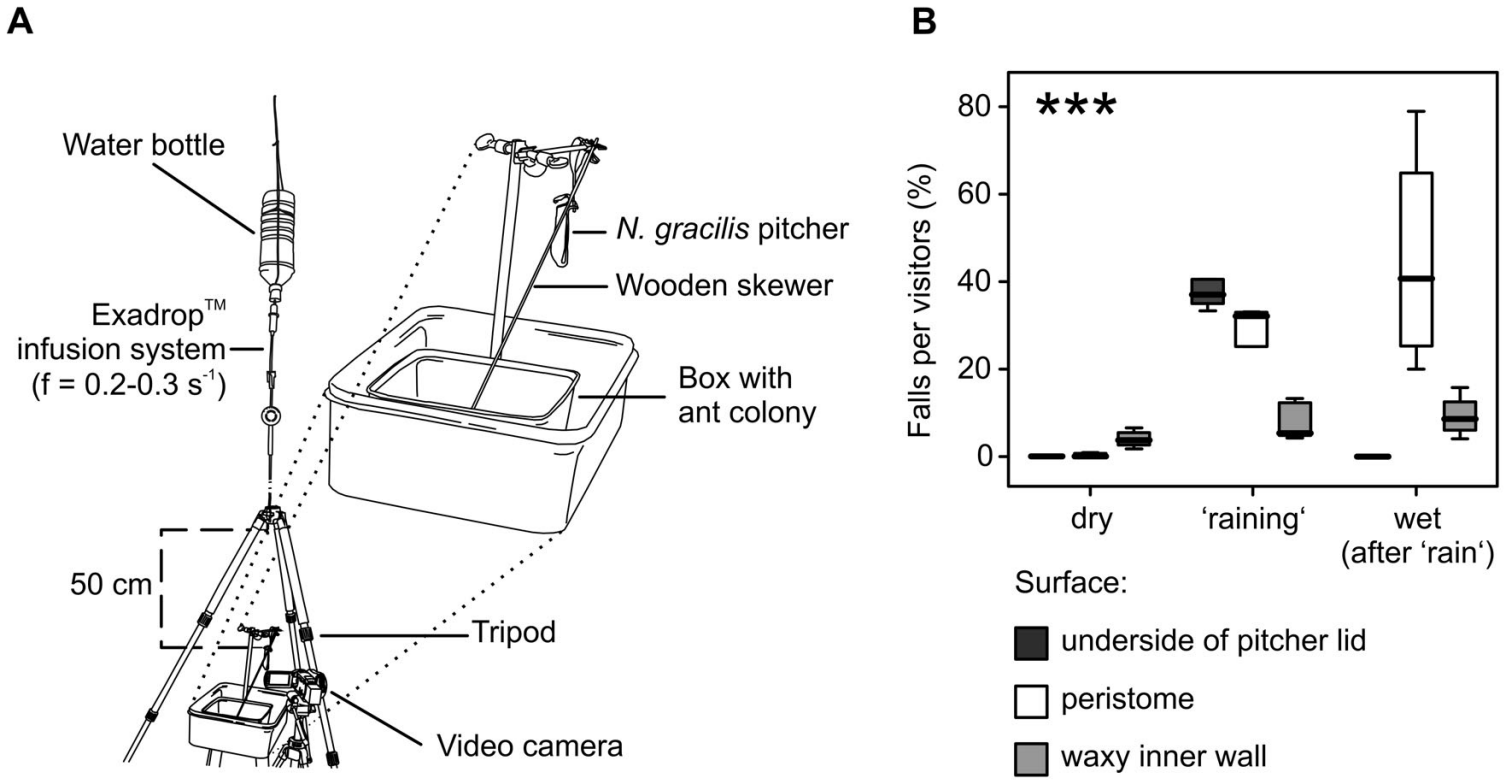
Contribution of the individual *N. gracilis* pitcher surfaces to prey capture under different environmental conditions. (A) Experimental setup to test how rain drops falling onto the pitcher lid affect ant capture. (B) Proportion of ant visitors to each pitcher surface that fell into the pitcher, under ‘dry’, ‘raining’, and ‘wet’ treatment condition, respectively. The interaction of pitcher surface and experimental condition was highly significant (*P*<0.001).

In the laboratory, we allowed *Crematogaster* sp. ants (a common prey species of *N. gracilis* at our study site) to forage on freshly harvested pitchers. Rain was simulated using an infusion drip system (Fig. 2A; see Methods). The effect was striking: 40.61±9.62% of all ants visiting the lower lid surface were knocked off by the impact of ‘rain’ drops and fell into the pitcher (Video S1). In contrast, not a single ant fell from the lid before or after the simulation of rain, confirming that the slipperiness of the lower lid surface was not altered by the increase of humidity after the ‘rain’. This result was not changed when an isolated pitcher lid (mounted horizontally using a paper clip) was tested: in this case 44% (11 of 25) ants were knocked off by ‘rain’ drops (Video S2). Ants were observed to be relatively ‘safe’ when holding onto the thicker mid-rib and get knocked off more frequently when they were positioned further out towards the (thinner) sides of the lid. Whether this was due to the mid-rib providing additional grip or to the dampened impact of the rain drops in this thicker section of the lid is not clear.

### *N. gracilis* pitchers rely on different trapping surfaces under different weather conditions

We investigated the contribution of each surface (inner wall, lower lid surface, peristome) under different experimental conditions (before/during/after simulated rain). We found a highly significant dependence of the surfaces' trapping efficiency on the experimental conditions (ANOVA comparing two separate Generalised Linear Mixed Models, for details see Methods, *df* = 4, χ^2^ = 185.97, *P*<0.001; Fig. 2B). Ants fell from the lower lid surface only under the impact of simulated ‘rain’ drops, in which case up to 57% of the visitors were captured. The (weather-independent) wax crystal layer on the inner pitcher wall provided a low but more or less constant baseline trapping efficiency (c. 7%). The peristome was not slippery when dry but reached high efficiency (up to 80%) under wet conditions. In our experiment, the peristome became slippery approximately 2–3 min after the start of the simulated rain, and stayed slippery for 7–10 min after we stopped the dripping.

### The lid of *N. gracilis* pitchers contributes to natural prey capture in the field

**Figure 3:**
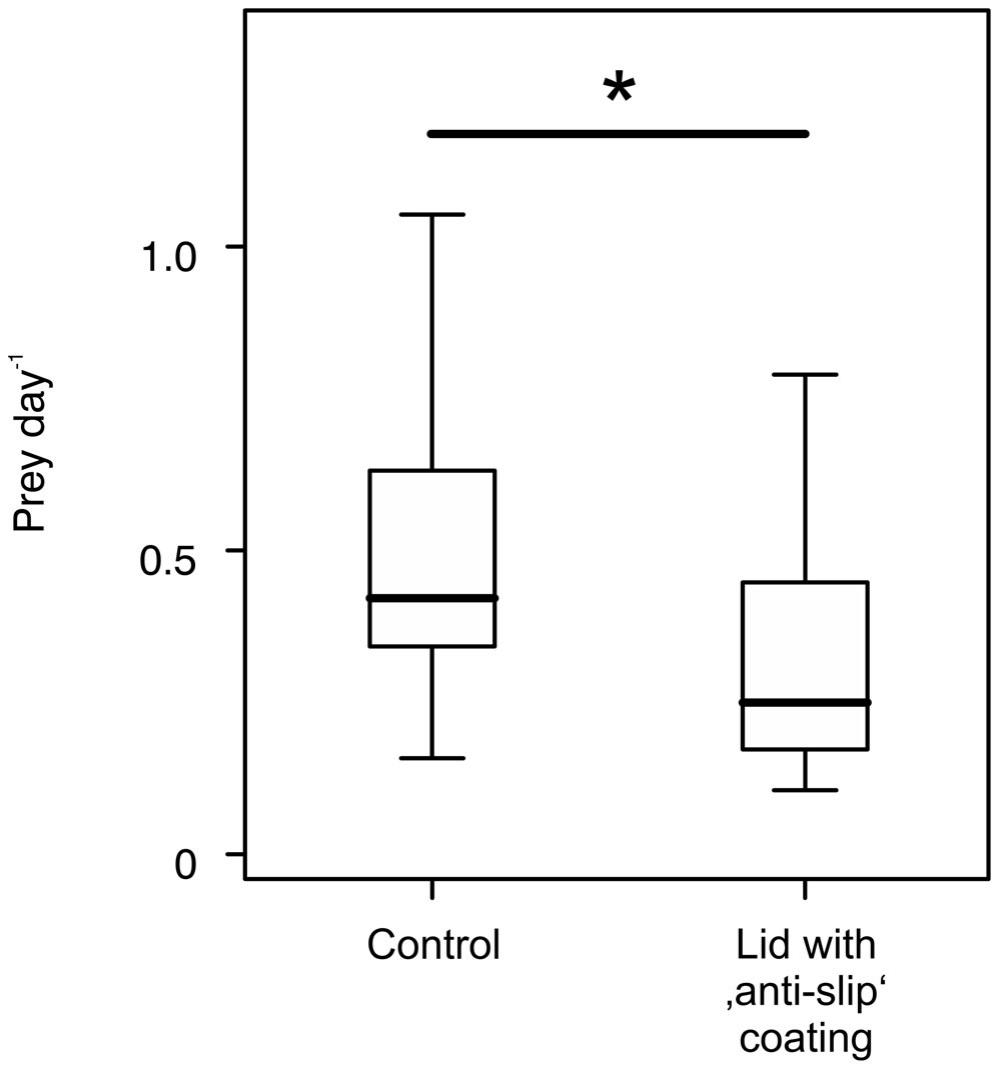
Biological relevance of the lid capture mechanism. The natural prey capture rate of pitchers with a non-slippery PDMS coating applied to the lower lid surface is reduced in comparison to the untreated control group (*: *P*<0.05).

We tested the biological relevance of the lid capture mechanism by comparing prey numbers between an untreated control and pitchers with experimentally modified lids (underside coated with a thin layer of a non-toxic, transparent silicon polymer). Ants are able to walk on this polymer surface under both wet and dry conditions. The ‘anti-slip coating’ of the lower lid surface caused a significant reduction of captured prey in the field (Mann-Whitney U test, *n_1/2_* = 15, *Z* = 1.97, *P*<0.05; Fig. 3). Prey numbers over the course of the experiment (19 days) were highly variable both between pitchers and between sampling intervals (3 days). Remarkably, the lid manipulation did not render pitchers completely ineffective: all pitchers did capture some prey over the course of the experiment. This indicates that the pitchers were still able to trap prey with the peristome and the inner wall.

### *N. gracilis* has evolved two structurally and functionally different wax crystal surfaces

**Figure 4:**
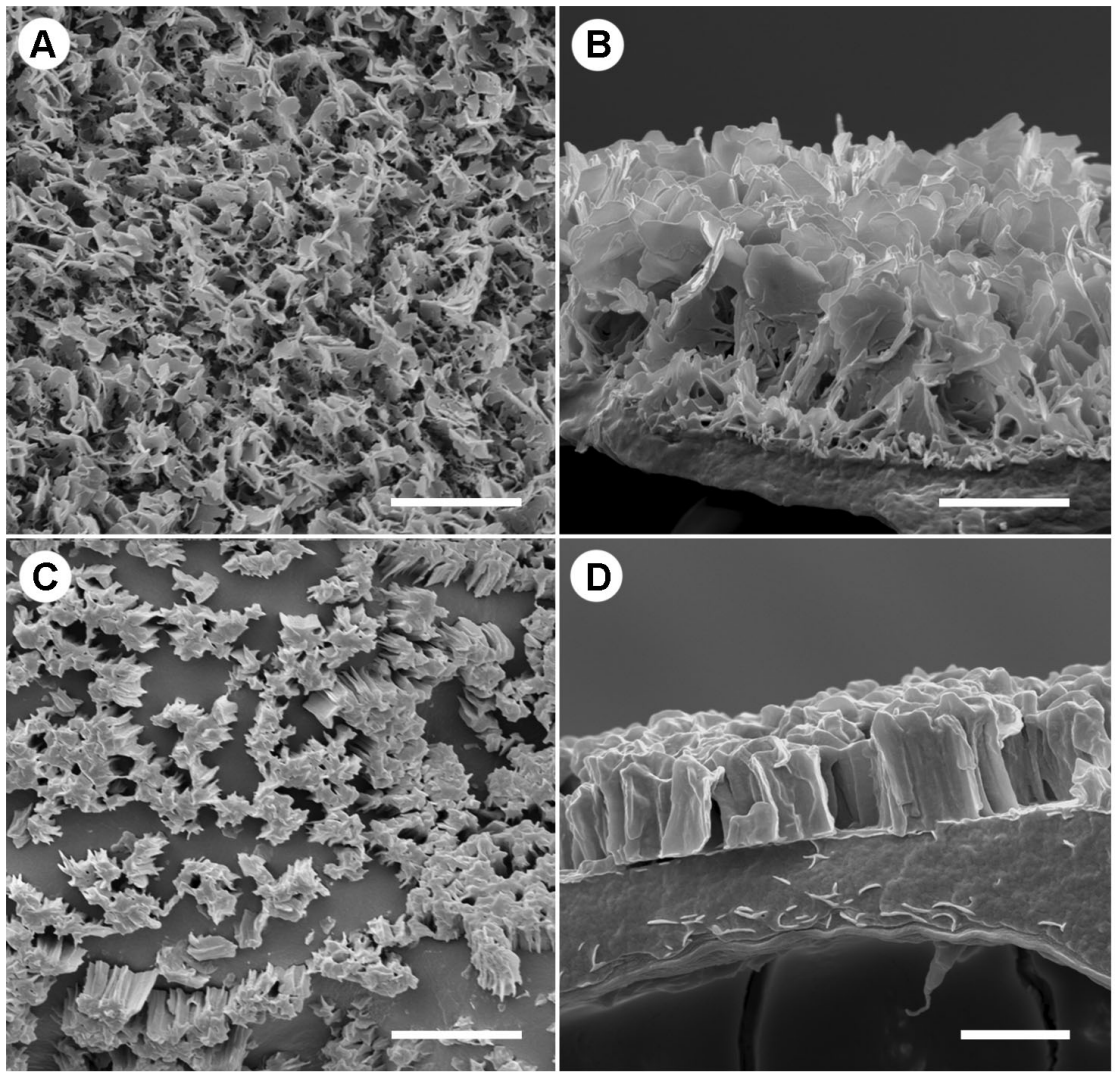
Microscopic structure of the wax crystal surfaces. (A–B) Microstructure of the crystalline wax layer on the inner pitcher wall. (A) Top view, showing a dense network of thin, upright wax platelets (scale bar: 5 µm). The freeze-fracture side view (B) reveals the internal organisation of the surface (scale bar: 2 µm). (C–D) Microstructure of the lower lid surface. (C) Top view: the wax crystals form solid, pillar-like structures, unevenly distributed across the surface and surrounded by smooth cuticle (scale bar: 5 µm). (D) Side view of the wax pillars (scale bar: 2 µm).

Scanning electron micrographs of the inner pitcher wall and underside of the pitcher lid revealed that both wax crystal surfaces are radically different in structure. The inner wall surface (Fig. 4A–B) was similar in morphology to wax crystal surfaces studied in other *Nepenthes* species, with a continuous, 3.05±0.36 µm (mean ± s.d., *n* = 21) thick layer of leaf-like wax platelets connected to an underlying matrix of shorter wax crystals [13], [17]. In contrast, the lower lid surface (Fig. 4C–D) was covered with discrete, pillar-like wax structures, 1.78±0.36 µm (mean ± s.d., *n* = 18) in height and 1.57 µm (median, range = 3.45 µm, *n* = 37) in diameter. The individual micropillars were unevenly distributed across the surface and sometimes densely clustered so that they appeared merged into solid blocks. The cuticular surface in between the micropillars was perfectly smooth and free of any crystal structures (Fig. 4C). The largest gaps between (clusters of) micropillars were typically 2.34±0.62 µm (mean ± s.d., *n* = 17) wide.

### Investment in prey attraction by *N. gracilis* is concentrated on the pitcher lid

**Figure 5:**
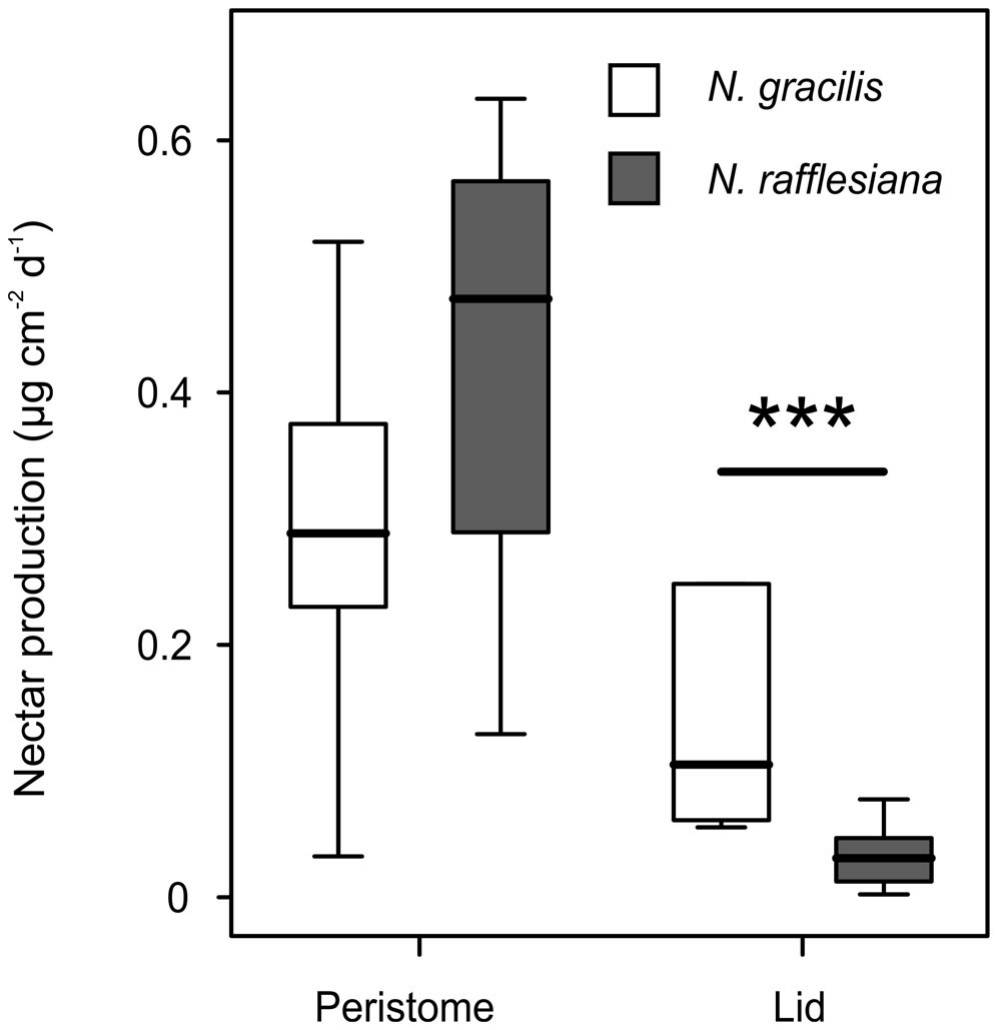
Area-specific nectar secretion onto the peristome and the lower lid surface. *N. gracilis* pitchers secrete significantly larger amounts of nectar under the lid than those of sympatric *N. rafflesiana* (***: *P*<0.001).

We compared the nectar production of *N. gracilis* and *N. rafflesiana* (without wax crystals under the lid) in the same field site, sampling every second day from both peristome and lower lid surface over a period of two weeks. The median area-specific daily amount of sugar secreted on the lower lid surface was 3.4 times higher in *N. gracilis* than in *N. rafflesiana* (Mann-Whitney U test, *n_1_* = 9, *n_2_* = 10, *Z* = 3.184, *P*<0.01; Fig. 5). In contrast, *N. rafflesiana* pitchers secreted slightly higher amounts of sugar onto the peristome; however, this difference was not statistically significant (Mann-Whitney U test, *n_1_*_/*2*_ = 10, *Z* = 1.285, *P* = 0.2).

## Discussion

The trapping function of the lower lid surface in *N. gracilis* constitutes a new trapping mechanism that has not been described previously. So far, the lid was only thought to play a role in prey attraction and as a protection against rain that would otherwise dilute the pitcher fluid [18]. The precarious position directly above the pitcher opening (Fig. 1B), however, makes the lower lid surface highly suitable as a trapping device. Our experiments have demonstrated the effectiveness of the lid trapping mechanism for ants, but we have observed it to work for beetles (Coccinellidae) and flies (*Musca domestica*) as well (Video S3).

The microroughness created by the wax pillars is likely to reduce the contact area between the ants' smooth adhesive pads and the lid surface [16]. However, some of the gaps between the micropillars may be large enough (≥3 µm) for ant claws to interlock [16], [19]. Furthermore, the compact, clustered arrangement of the micropillars should render them less likely to break under the claw-induced stress. Effective claw use may explain the observed good walking performance of ants in the absence of rain impact.

Intermittent slipperiness of the peristome has been suggested as a strategy to ensure the survival of ‘scout’ ants and promote subsequent recruitment or worker ants to the pitcher. The lid capture mechanism may have a similar effect on the recruitment of social insects to the nectaries under the lid. Rainfalls in the distributional range of *N. gracilis* (Borneo, Sumatra, Malay Peninsula and central Sulawesi [18]) are typically brief and heavy with intensities of up to over 90 mm h^−1^ and most rain falling within less than one hour [20], [21]. The lower lid surface is therefore a safe place to forage for most of the time. The marginally significant reduction of trapping success by the ‘anti-slip coating’ on the lower lid surface, despite the presence of other effective trapping mechanisms, indicates an important contribution of the lid towards natural prey capture in *N. gracilis*. It is currently not clear whether this mechanism is a unique feature of *N. gracilis* or a more widespread phenomenon in *Nepenthes*. A similar manipulation of the lid in *N. rafflesiana* pitchers in a recent study did not show an effect on prey capture [4], and wax crystals are absent from the lower lid surface of this species. Thus, it is likely that the wax micropillars are crucial for the trapping function of the lid in *N. gracilis*.

Our results suggest that *N. gracilis* has not only evolved special morphological adaptations to capture prey with the pitcher lid, but has also adjusted its nectar secretion patterns to increase prey attraction to the lower lid surface. The peristome of *N. gracilis* pitchers, although fully functional (Fig. 2B), is very narrow, and larger insects can easily use their claws to hold on to its outer edge while harvesting nectar (Fig. 1A). It has recently been demonstrated that many *Nepenthes* species have specialised to prioritise either the peristome or the inner wall for trapping, manifested in two extreme pitcher morphologies: strongly enlarged peristomes and smooth inner walls on one hand, and narrow peristomes and well-developed wax crystal layers on the other [5]. *N. gracilis* has further specialised by evolving a new type of wax crystal surface under the pitcher lid. These wax crystals appear to provide the right level of slipperiness to cause insects to fall into the pitcher when the lid vibrates while allowing them to approach the nectaries safely at other times. Further experiments and field studies should be conducted to elucidate the detailed biomechanical underpinnings of this new trapping mechanism, and to investigate what implications it has for the prey spectrum of *N. gracilis*.

## Materials and Methods

### Ethics Statement

Permission to conduct field research in Brunei Darussalam was granted through the appointment of UB as a research associate with University Brunei Darussalam. No further permits were required as the study was conducted on publicly owned, not protected land. *N. gracilis* and *N. rafflesiana* are not protected under Brunei law. CITES export (No. BA/MAP/02/1003) and import (No. 358814/05) permits were obtained to export plant material for SEM analysis.

### Plant material and field site

The study was conducted in the Tutong district of Brunei, Northern Borneo, in July 2011. Our field site was a heavily degraded roadside habitat with open, shrub-dominated vegetation over white silica sands. Both *N. gracilis* and *N. rafflesiana* are highly abundant in this site while the hybrid *N. gracilis* × *N. rafflesiana* is rare. All experiments were performed on upper pitchers of *N. gracilis*, using either live pitchers in the field, or freshly collected pitchers in the laboratory. Upper pitchers of *N. rafflesiana* were used for comparison in the measurements of nectar production. Plant material for SEM analysis was obtained from the field (two pitchers from different plants) and from the Royal Botanic Gardens of Kew (three pitchers from two different clones/three individual plants).

### Effect of simulated rain on the capture efficiency of *N. gracilis* pitchers

Five upper pitchers were tested under three different conditions: dry, during and directly after simulated rain. Each pitcher was collected from the field immediately (<30 min) before the start of the experiment by cutting the leaf at the base. In the laboratory, the leaf was fixed to a tripod stand to simulate the natural orientation of both leaf and pitcher. A colony of *Crematogaster* sp. ants was collected from the same field site three days in advance and kept in a plastic container. The ants were given access to the experimental pitcher via a wooden skewer and immediately started to recruit workers to forage on the secreted nectar.

Rain was simulated experimentally using an Exadrop™ drip infusion system with a precision flow control (B. Braun, Melsungen) attached to a 1.5-litre plastic bottle with distilled water. The outlet of the drip tube was fixed to a standard photographic tripod and positioned in 50 cm distance directly above the pitcher lid (Fig. 2A). The drip frequency was adjusted to 0.22–0.32 s^−1^. The simulated rain drops had a mass of 38–44 mg (range of *n* = 60 droplets), corresponding to a spherical drop diameter of 4.2–4.4 mm, and reached a velocity of 3.0±0.3 m s^−1^ (mean ± s.d. from *n* = 10 droplets measured using an A-602f Basler camera at 304 frames per second) which is roughly one third of the terminal velocity for that drop size [22]. For comparison, most rain drops in tropical rains are typically between 1.5 and 3 mm in diameter, and frequently reach >4 mm at the leading edge of storms or after interception by vegetation [23], [24]. The impact momentum of our simulated droplets (0.125 g m s^−1^) is within the range of natural rain (0.028–0.60 g m s^−1^) [25].

A digital video camera (Sony DCR-SR35E) was positioned in front of the pitcher so that a full size view of the peristome and the underside of the pitcher lid could be recorded. The foraging ants were observed and videotaped for a total of 30 min on each pitcher, 10 min each before, during and directly after simulated rain. Videos were analysed by counting the number of falls from each surface (underside of the lid, peristome, and inner pitcher wall) in relation to the number of visitors on the respective surface. Since the inner pitcher wall bears no nectaries it is not normally visited by foraging ants, but ants foraging on the peristome nectaries occasionally stray out onto the inner wall and get trapped. We therefore counted the number of falls from both peristome and inner pitcher wall in relation to the number of visitors on the peristome.

An additional experiment was performed on an isolated *N. gracilis* lid that was fixed in natural horizontal orientation on a tripod using a paper clip. *Crematogaster* sp. ants were given access to the lid as described above for the whole pitcher. We videotaped the performance of the ants on the lower lid surface while simulating rain with the above described drip method.

### Anti-slip coating of the lower lid surface of naturally growing pitchers

Thirty *N. gracilis* pitchers (each on a different plant) were labelled in the field and randomly assigned to an experimental or a control group. Using a fine paint brush, a thin layer of a non-toxic, transparent and odourless PDMS polymer (Sylgard™ 184, Dow Corning, Midland) was applied to the lower lid surface of the pitchers in the experimental group. Sylgard™ 184 has been shown to have no measurable effect on insect attraction but provide a hydrophobic, non-slippery surface for insects [4].

All prey was removed from the pitchers and the fluid was filtered through a Nuclepore™ track edge membrane filter (25 mm diameter, 12 µm pore size, Dow Corning). A small polyurethane cone (cut from a commercial ear plug) was inserted into the tapered bottom end of the pitcher to prevent the loss of prey. Prey was sampled every third day for a total of 19 days by sucking out the pitcher fluid using a 10 mL syringe with an attached silicone tube, transferring the fluid to a petri dish, and removing all prey manually with a pair of fine spring steel tweezers.

### Comparison of the wax crystal structure on the lid and the inner pitcher wall

Five *N gracilis* upper pitchers were collected in airtight plastic bags and transported in a cool box to the laboratory where they were quench-frozen (3–4 hours after collection) in liquid propane cooled in liquid nitrogen. Approximately 1 cm^2^ pieces of the pitcher lid and inner pitcher wall were cut with a razor blade, freeze-dried, mounted on SEM stubs and sputter-coated with a 20 nm layer of gold. Alternatively, samples were freeze-fractured before freeze-drying. The microstructure of the wax crystal layers on the lower lid surface and inner wall surface was examined using a Philips FEI XL30-FEG SEM with an accelerating voltage of 5.0 kV.

### Measurement of nectar production in the field

In a plot of approximately 15×50 m, 10 pitchers each of *N. gracilis* and *N. rafflesiana*, each on a different plant, were labelled and roofed with transparent sheets of stiff plastic foil to protect the nectar from being washed off by rain. To prevent insects from collecting the nectar, we applied sticky Tangletrap™ resin to the base of the leaf and enclosed each pitcher in a fine-mesh gauze bag. At the start of the experiment, we removed all nectar from the peristome and lower lid surface by repeatedly wiping the surface with approximately 1 cm^2^ sized squares of wet laboratory wipe (Kimwipe™, Kimberley-Clarke, Reigate) held with a pair of self-closing blunt forceps.

Nectar from both the peristome and the lower lid surface was sampled every second day over a period of two weeks. Samples were obtained by moistening the surface with a wet Kimwipe™ square and then wiping it with a dry piece of a highly absorbent medical swap (Sugi™, Kettenbach Medical, Eschenburg) made from cotton and cellulose. Individual Sugi™ swaps were cut into 3–4 small pieces to minimise the use of absorbent material, and were handled using forceps and latex gloves to avoid contamination. Samples from the peristome and from the lid of both *Nepenthes* species were collected separately in Eppendorff tubes and dried over silica gel for 5–7 days. The completely dried samples were re-diluted in the smallest possible amount of distilled water (between 0.1 and 0.6 mL) depending on the amount of absorbent material used, and the sugar content was measured with a handheld refractometer (ATAGO, L. Kübler, Karlsruhe).

The measured sugar secretion was corrected for the varying area of the sampled surfaces to allow for a direct comparison between the two *Nepenthes* species regardless of their different pitcher size and geometry. Surface areas were measured after the final nectar sampling by mounting the surfaces flat (cut in smaller pieces where necessary) on graph paper, pressing them down with a glass plate, and taking a photograph from above. The areas were then measured digitally using Scion Image (release Alpha 4.0.3.2, Scion Corporation, Frederick) software.

### Statistical analysis of data

Statistical tests were conducted using the software packages BiAS. for Windows and R. Data were tested for normality using Shapiro-Wilks tests, and non-parametric tests were used were appropriate. Throughout the paper, descriptive statistics denote mean ± s.d. for normally distributed data and median and range in all other cases. Effects were considered significant when *P*<0.05.

To analyse the results of the rain simulation experiment, General Linear Mixed Models (GLMMs, appropriate for proportional count data) were fitted to the data. The experimental conditions (before/during/after simulated rain) and the individual surfaces (peristome, lower lid surface, inner pitcher wall) were considered fixed factors. To improve the accuracy of the model, ‘surface’ (nested in the random factor ‘pitcher’) was also included as a nested random factor. We calculated two separate GLMMs, with and without interaction of the fixed factors. In a second step, a conventional one-way ANOVA was performed to compare both models: significant differences between the models indicate a significant fixed factor interaction.

## Supporting Information

Video S1 **Effect of simulated rain on ants foraging on the underside of the pitcher lid of** ***N. gracilis.***
(AVI)

Video S2 **Effect of simulated rain on ants foraging on the underside of an isolated** ***N. gracilis*** **pitcher lid.**
(AVI)

Video S3 **High-speed video recording (recording frame rate: 428 s^−1^, playback frame rate: 10 s^−1^) of a house fly (** ***Musca domestica*** **) being knocked off the underside of an** ***N. gracilis*** **lid by a simulated rain drop and captured.**
(AVI)
